# Whole Genome Sequencing Identifies Key Genes in Spinal Schwannoma

**DOI:** 10.3389/fgene.2020.507816

**Published:** 2020-10-30

**Authors:** Xin Gao, Li Zhang, Qi Jia, Liang Tang, Wen Guo, Tao Wang, Zheyu Wu, Wang Zhou, Zhenxi Li, Jianru Xiao

**Affiliations:** ^1^Orthopedic Oncology Center, Department of Orthopedics, Changzheng Hospital, Second Military Medical University, Shanghai, China; ^2^Key Laboratory of Advanced Theory and Application in Statistics and Data Science – MOE, School of Statistics, East China Normal University, Shanghai, China; ^3^Center for Bioinformatics and Computational Biology, School of Life Sciences, Institute of Biomedical Sciences, East China Normal University, Shanghai, China; ^4^Department of Orthopedics, Taizhou People’s Hospital, Taizhou, China; ^5^Department of Orthopedics, Zhongnan Hospital of Wuhan University, Wuhan, China

**Keywords:** hippo signaling pathway, copy number alterations, frequently mutated genes, whole genome sequencing, spinal schwannoma

## Abstract

Spinal schwannoma is the most common primary spinal tumor but its genomic landscape and underlying mechanism driving its initiation remain elusive. The aim of the present study was to gain further insights into the molecular mechanisms of this kind of tumor through whole genome sequencing of nine spinal schwannomas and paired blood samples. The results showed that *ATM, CHD4, FAT1, KMT2D, MED12, NF2*, and *SUFU* were the most frequently mutated cancer-related genes. In addition, the somatic copy number alterations (CNA) was potentially associated with spinal schwannoma, among which *NF2* was found to be frequently deleted in schwannoma samples. Only a few genes were located within the amplified regions. In contrast, the deleted regions in 15q15.1 and 7q36.1 contained most of these genes. With respect to tumorigenesis, *NF2* had the highest variant allele frequency (VAF) than other genes, and homozygous deletion was observed in *NF1*, *NF2*, and *CDKN2C*. Pathway-level analysis suggested that Hippo signaling pathway may be a critical pathway controlling the initiation of spinal schwannoma. Collectively, this systematic analysis of DNA sequencing data revealed that some key genes including *NF1*, *NF2*, and *CDKN2C* and Hippo signaling pathway were associated with spinal schwannoma, which may help improve our understanding about the genomic landscape of spinal schwannoma.

## Introduction

Spinal schwannoma is the most common primary spinal tumor, accounting for almost one-third of all spinal tumors (Seppala et al., 1995; Abul-Kasim et al., 2008), with an annual incidence of 0.3 to 0.4 per 100,000 (Seppala et al., 1995). Patients with spinal schwannoma usually have to endure pain, spinal root deficits, pyramidal tract compression, and sphincter disorders, which negatively affect patients’ quality of life (Lenzi et al., 2017). The gold standard treatment for spinal schwannomas is complete surgical resection (Jinnai and Koyama, 2005; Safaee et al., 2017). However, complete tumor removal is associated with a high incidence of complications, in which cutting the involved functionally relevant nerve root is likely to result in a permanent and significant neurological deficit (Celli et al., 2005), with up to 30% of patients developing postoperative neurologic deteriorations (Safaee et al., 2017). Unfortunately, there are no medical therapies available for spinal schwannomas, while a safe and effective treatment for these patients is urgently needed (Campian and Gutmann, 2017). Therefore, it is of great importance to gain a comprehensive insight into the genetic landscape of schwannoma and to identify the potential genes driving its initiation, thereby seeking therapeutic targets.

Schwannomas are derived from Schwann cells, the myelin-producing cells of the peripheral nervous system. Schwannomas can occur either spontaneously or as the hallmark tumor of neurofibromatosis type 2. Previous studies have identified several schwannoma-predisposing mutations. Specifically, mutation of the *NF2* gene is the most characteristic genetic risk factor for schwannoma (Rouleau et al., 1993; Trofatter et al., 1993; Lin and Gutmann, 2013; Pathmanaban et al., 2017; Carlson et al., 2018; Roberts et al., 2019). Germline mutations in *LZTR1* predispose to an inherited disorder of multiple schwannomas (Piotrowski et al., 2014). Mutation of the *SMARCB1* gene was found in 45% of familial and 7% of sporadic schwannomatosis cases (Smith et al., 2012), and it was also reported to play a role in the development of some sporadic spinal schwannomas (Paganini et al., 2018). In order to determine the frequency of these predisposing mutations in solitary schwannomas, Pathmanaban et al. screened 135 schwannoma cases using Sanger sequencing or next-generation sequencing, and the results showed that *NF2*, *LZTR1*, or *SMARCB1* mutations were found in 54.5% of them (Pathmanaban et al., 2017). Furthermore, in an integrative analysis performed by Agnihotri et al. (2016) whole exome sequencing analysis identified recurrent mutations in *NF2*, *ARID1A*, *ARID1B*, and *DDR1*, and RNA sequencing identified a recurrent in-frame *SH3PXD2A-HTRA1* fusion in 10% cases. Whole exome sequencing was also performed on vestibular schwannomas. *NF2*, *CDC27*, and *USP8* were identified to be the most common tumor-specific mutations (Havik et al., 2018). Nevertheless, a focused attention on the genomic landscape of schwannomas in the spine is still needed. Furthermore, mutations in non-coding regions, such as introns, regulatory elements, and non-coding RNA, remain widely unexplored.

In this study, whole genome sequencing analysis of nine paired spinal schwannomas (ICD-O of 9560/0) and blood samples was performed to provide some useful information regarding the overall landscape of driver mutations and mutated pathways in schwannomas genomes.

## Materials and Methods

### DNA Extraction and Whole Genome Sequencing

This study was approved by the Changzheng Hospital medical ethics committee, and genomic DNA samples were obtained from fresh tissues and blood samples after being resected during surgery. All the patients were recruited with written informed consent, in accordance with the Declaration of Helsinki. TIAN amp Blood DNA Kit (TIANGEN Biotech Co., Ltd., Beijing, China) was used for extracting genomic DNA from paired schwannoma and blood samples according to the manufacturer’s instructions. The whole genome DNA were sequenced by Illumina X-Ten platform in Shanghai, China, and 150 bp paired-end reads were generated.

### Reads Mapping and Variants Calling and Annotation

The whole genome paired reads of 300bp (150bp at each end) were mapped to human reference genome (UCSC hg19 assembly) using BWA 0.7.12 “mem” mode with default options (Li and Durbin, 2009). The PCR duplicates of the mapped reads and low-quality reads (BaseQ < 20) were then removed by SAMtools “rmdup” with version 0.1.19. The resulting bam files were sorted and indexed by SAMtools sort and index, respectively. Somatic mutations were called by Strelka 2.8.4 software (Saunders et al., 2012) with default options. The somatic mutations should have a minimal of 4 read counts supporting the variant and over 20 reads covering the locus. The somatic copy number alteration (CNA)s were called by SAASCNV 0.3.4 (Zhang and Hao, 2015) with *P*-value < 0.05. We used the ANNOVAR software for variant annotation (Wang et al., 2010). It should be noted that all the somatic mutations were identified in the tumor tissues but absent in the corresponding normal tissues.

### Analyses of the Somatic Mutations in Schwannoma

The analyses included identification of potential genes driving schwannoma initiation, and identification of frequently mutated pathways. The potential genes driving schwannoma initiation were identified based on the variant allele frequency (VAF), and the frequently mutated pathways were identified based on the number of mutated genes in the oncogenic pathways. All the analyses were implemented in R maftools package (Mayakonda et al., 2018). The clonality analysis was conducted in R package CLONETv2 with default options (Carreira et al., 2014).

### Significantly Amplified and Deleted Regions

The somatic CNAs were first segmented by SAASCNV (Zhang and Hao, 2015). The significantly amplified and deleted regions were identified by GISTIC 2.0 on the Gene Pattern webserver (Mermel et al., 2011). The CNAs with *q*-value < 0.05 were deemed as the significantly amplified and deleted regions.

### Gene Set Enrichment Analysis

The gene sets collected from KEGG pathways were used in the enrichment analysis. The gene set enrichment analysis (GSEA) was conducted by hypergeometric test. The GSEA was implemented in R clusterProfiler package (Yu et al., 2012). The pathways were considered statistically significant if the *q*-value < 0.05.

## Results

### Clinical Characteristics and Outcomes of the Patients

The characteristics of 9 patients are shown in Table 1. This cohort was comprised of 4 men and 5 women, with a mean age of 57.6 years (median 63, range 27–69). The most common symptom was pain and hypesthesia. One patient (case 8) was diagnosed with the recurrent schwannoma which was totally resected 15 years ago. Tumor size ranged from 1 to 6.5 cm in the maximum diameter, with 2 of them larger than 5 cm. Schwannomatosis were diagnosed in 3 patients who had multiple spinal schwannomas without bilateral vestibular schwannoma. No pathogenic germline NF2 mutations were found in these patients. Complete resection of the tumor(s) and posterior stabilization of the spine was performed for all patients. According to postoperative pathological examination, the tumor cell percentages of all these samples were above 88% (Range 88–95%). Postoperatively, all patients recovered well without surgical complications. The mean follow-up duration was 42.9 (median 45, range 33–48) months. All patients were alive with no evidence of disease at the last follow-up in July 2019.

**TABLE 1:** Clinical characteristics of nine patients with spinal schwannoma.

**No.**	**Sex**	**Age**	**Location**	Single/multiple*	**Primary/recurrent**	**Histological grade**	**Tumor size (cm)**	**Adjacent relations to spinal canal**	**Shape**	**Bone structure destruction**	**Resection mode**	**Follow-up (M)**	**Final Status**
1	M	47	L1-2	Multiple	Primary	Grade I	2.5; 1.5; 1	Intraspinal	Round	No	Total	35	NED
2	M	66	L2; S1	Multiple	Primary	Grade I	2.4; 1.8	Intraspinal	Round	No	Total	47	NED
3	F	65	C6-7	Single	Primary	Grade I	6	Intra-extraspinal	Irregular	Yes	Total	33	NED
4	F	53	L2	Single	Primary	Grade I	2.4	Intraspinal	Ellipse	No	Total	48	NED
5	M	62	S1-2	Single	Primary	Grade I	3.5	Intra-extraspinal	Round	yes	Total	43	NED
6	F	63	T1-2	Single	Primary	Grade I	4.3	Intra-extraspinal	Dumbbell	No	Total	46	NED
7	M	27	L4-5	Single	Primary	Grade I	6.5	Intra-extraspinal	Dumbbell	Yes	Total	45	NED
8	F	66	L1-2	Single	Recurrent	Grade I	4.2	Intraspinal	Ellipse	No	Total	44	NED
9	F	69	L3-4	Multiple	Primary	Grade I	2; 1.1	Intraspinal	Round	No	Total	45	NED

NED, no evidence of disease. *The three patients with multiple spinal schwannomas were diagnosed as schwannomatosis. They were sporadic schwannoma(s) without family history.

### Whole Genome Sequencing of Nine Spinal Schwannomas and Paired Blood Samples

**FIGURE 1:**
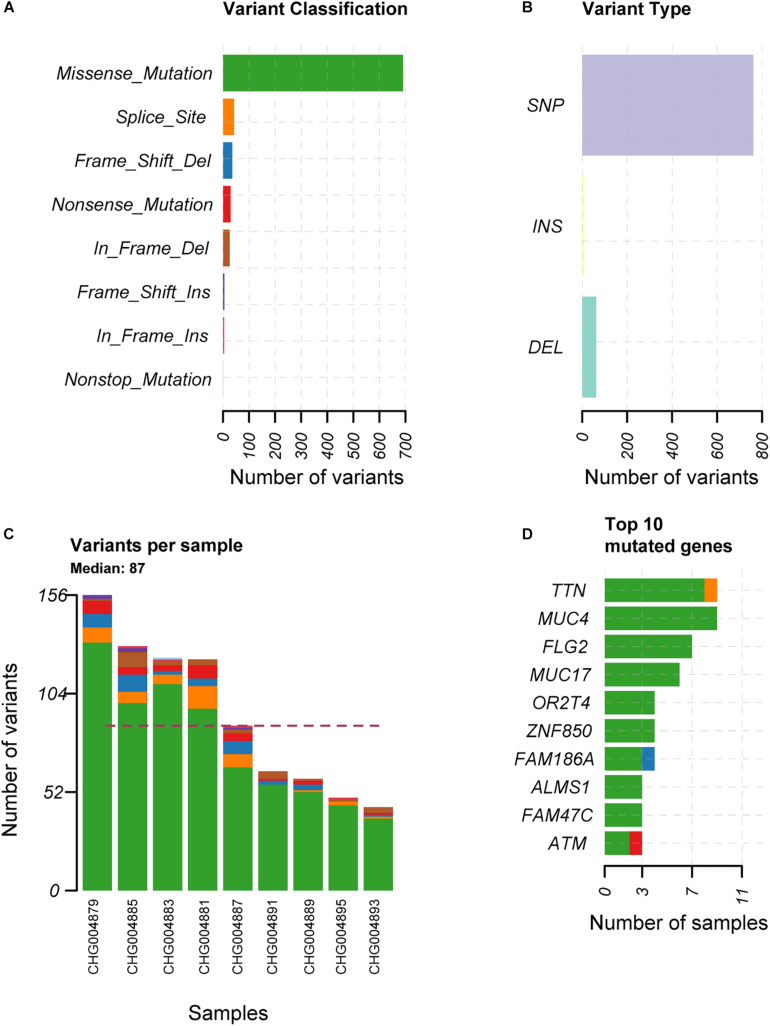
The overview of the somatic mutations in spinal schwannoma. The variant classification, variant type, the number of variants per sample, and the top-ten mutated genes in the nine spinal schwannomas are displayed in **(A–D)**, respectively. The *x*-axis in **(A,B,D)** represent the number of mutations or samples. The *x*-axis in **(C)** represents the nine samples.

To explore the alterations in the genome of spinal schwannoma, we performed whole genome sequencing (WGS) to a median depth of 36.77X (range, 32.34X to 40.59X), and identified 832 somatic mutations, which were present in tumors but absent in paired blood samples, including 763 single nucleotide variants (SNVs) and 69 insertion or deletions (InDels) across the 9 patients (Figures 1A,B), with a median of 87 variants (Figure 1C). Based on the RefSeq gene annotation, we identified *TTN*, *MUC4*, *FLG2*, *MUC17*, *OR2T4*, *ZNF850*, *FAM186A*, *ALMS1*, *FAM47C*, and *ATM* as the top ten mutated genes (Figure 1D), however, only *ATM* was previously reported to be implicated in cancer (Kim et al., 2014; Chen et al., 2015; Feng et al., 2015).

### Genetic Landscape of Spinal Schwannoma

**FIGURE 2:**
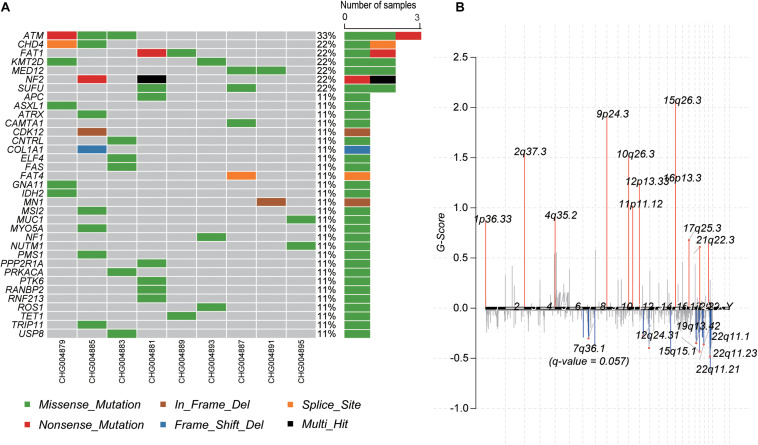
The mutational landscape of spinal schwannoma. **(A)** The somatic mutations of cancer driver genes across the nine spinal schwannomas. **(B)** The somatic copy number alterations along the chromosomes. The G-score was to evaluate the significance of the gains and losses.

To identify the genes responsible for schwannoma, we further investigated whether the mutated genes could be found in the COSMIC Cancer Gene Census database (Forbes et al., 2008), a database curating genes causally implicated in cancer. As shown Figure 2A, *ATM*, accounting for 33% of the samples, was the most frequently mutated cancer-related gene in schwannoma, followed by *CHD4* (22%), *FAT1* (22%), *KMT2D* (22%), *MED12* (22%), *NF2* (22%), and *SUFU* (22%). Most of them were predicted as pathogenic by SIFT, PolyPhen-2, or MutationTaster (Table 2). Particularly, *NF2* (Neurofibromin 2) has been widely identified as the pathogenic genes for both sporadic and familial schwannoma (Jacoby et al., 1994; Rm et al., 2018).

**TABLE 2:** The pathogenicity of the somatic mutations in spinal schwannoma by SIFT, PolyPhen-2, and MutationTaster.

**Gene Symbol**	**Effect**	**Transcript:exon:CDS**	**SIFT**	**Polyphen2-HDIV**	**Polyphen2-HVAR**	**Mutation Taster**	**Sample**	**VAF**
ATM	Non-sense	NM_000051:exon46:c.C6725A:p.S2242X	NA	NA	NA	D	CHG004878	0.13
ATM	Missense	NM_000051:exon60:c.T8699C:p.L2900P	D	D	D	D	CHG004882	0.13
ATM	Missense	NM_000051:exon24:c.T3458C:p.V1153A	T	B	B	N	CHG004884	0.10
CHD4	Missense	NM_001297553:exon9:c.C1460T	T	D	B	D	CHG004878	0.13
CHD4	Missense	NM_001297553:exon18:c.G2831A:p.G944E	D	D	D	D	CHG004884	0.34
FAT1	Non-sense	NM_005245:exon2:c.G370T:p.E124X				A	CHG004880	0.14
FAT1	Missense	NM_005245:exon10:c.C6110T:p.T2037M	D	B	B	N	CHG004888	0.18
KMT2D	Missense	NM_003482:exon34:c.G9484A:p.G3162S	D	B	B	N	CHG004878	0.10
KMT2D	Missense	NM_003482:exon48:c.G15713A:p.R5238Q	T	D	P	D	CHG004892	0.14
MED12	Missense	NM_005120:exon3:c.C385A:p.L129I	D	D	D	D	CHG004886	0.25
MED12	Missense	NM_005120:exon28:c.G4037A:p.R1346H	D	P	B	D	CHG004890	0.25
NF2	Missense	NM_181830:exon14:c.G1517A:p.C506Y	D	D	D	D	CHG004880	0.22
NF2	Non-sense	NM_181830:exon10:c.C979T:p.Q327X				A	CHG004884	0.50
NF2	Inframe deletion	NM_181830:exon6:c.513_557del:p.171_186del	NA	NA	NA	NA	CHG004880	0.53
SUFU	Missense	NM_001178133:exon10:c.C1177T:p.R393W	D	D	D	D	CHG004880	0.49
SUFU	Missense	NM_001178133:exon6:c.G691A:p.G231S	D	D	D	D	CHG004886	0.11

*D, deleterious; P, probably deleterious; B, benign; T, tolerant; A, disease causing automatic; N, polymorphism; NA, not available.*

Moreover, we also profiled the somatic CNA in schwannoma based on the GISTIC algorithm (*q*-value < 0.05). The identified significantly amplified regions were 1p36.33, 2q37.3, 4q35.2, 9p24.3, 10q26.3, 11p11.12, 12p13.33, 15q26.3, 16p13.3, and 17q25.3, while the significantly deleted regions were 12q24.31, 15q15.1, 19q13.42, 22q11.1, 22q11.21, and 22q11.23 (Figure 2B). In accordance with the somatic mutations and InDels, *NF2*, harbored in the cytoband 22q11, was also identified to be frequently deleted in the samples of schwannoma (Supplementary Figure S1). These results indicated that *NF2* played a key role in the initiation of schwannoma.

Furthermore, we also investigated the genes within these CNA regions potentially involved in spinal schwannoma. Only a few genes were located within the amplified regions. whereas the deleted regions contained most of these genes (Supplementary Tables S1, S2). Specifically, *ITPKA*, *LTK*, *CHP*, *OIP5*, *RTF1*, *RPAP1*, *NDUFAF1*, *NUSAP1*, *INO80*, *EXD1*, and *OIP5-AS1*, fell within the cytoband of 15q15.1 (*n* = 6, 66.7%). The gene set enrichment analysis revealed that these genes might participate in the maintenance of chromosomal structure, such as DNA conformation change, chromosome segregation, and nucleosome organization (*q*-value < 0.05). In addition, we observed that *XRCC2* and *MLL3/KMT2C* were located within 7q36.1 (*n* = 5, 55.6%), which had a *q*-value 0.057, slightly higher than the threshold 0.05, however, the two genes were involved in the tumorigenesis or progression of several malignant tumors (Figure 2B and Supplementary Table S1), suggesting that these genes might play key roles in schwannoma.

### The Potential Genes Driving Schwannoma Initiation

**FIGURE 3:**
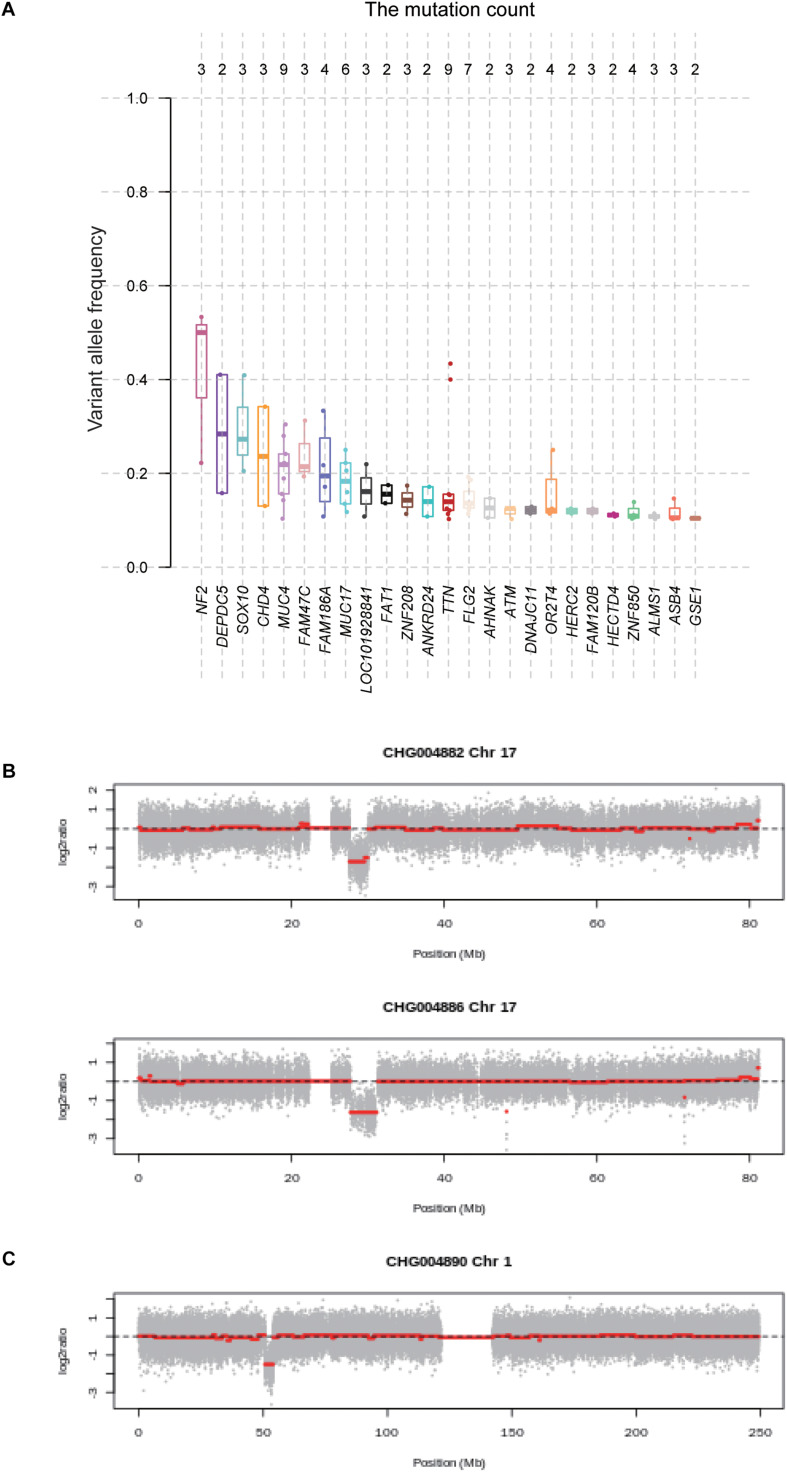
The variant allele frequency (VAF) of the genes mutated in spinal schwannomas. **(A)** The numbers on the top represent the number of mutations. The genes are ordered by the median of VAF across the mutations. **(B,C)** The log2 copy number ratio of chromosomes 17 and 1 in the spinal schwannoma samples.

With the whole genome sequencing data, we aimed to identify the genes potentially driving schwannoma initiation. In tumorigenesis, the more the VAF was close to 50%, the earlier the mutation may occur. We then ranked the genes based on the median of VAF across the samples (Figure 3A). Among the genes mutated in more than one sample, *NF2* had a higher VAF than other genes, followed by *DEPDC5*, which were involved in mTOR signaling pathway. Furthermore, we also conducted the subclonality analysis, and identified that *NF2* gene mutations were present in all subclones of CHG004880 and CHG004884, suggesting that *NF2* was a candidate driver in spinal schwannoma (Supplementary Figure S2). Particularly, *SOX10* and *CHD4* also showed relatively high VAF among those genes (VAF > 20%). In addition, we also examined the five samples without *NF2* gene mutations. Interestingly, the spinal schwannoma samples of CHG004882 and CHG004886 harbored homozygous deletion in *NF1* (Figure 3B). Homozygous deletion of CDKN2C was observed in CHG004890 (Figure 3C). These results indicated that the CNV loss in *NF1* and *CDKN2C* might also contribute to tumorigenesis of spinal schwannoma.

### Identification of Frequently Mutated Pathways in Schwannoma

**FIGURE 4:**
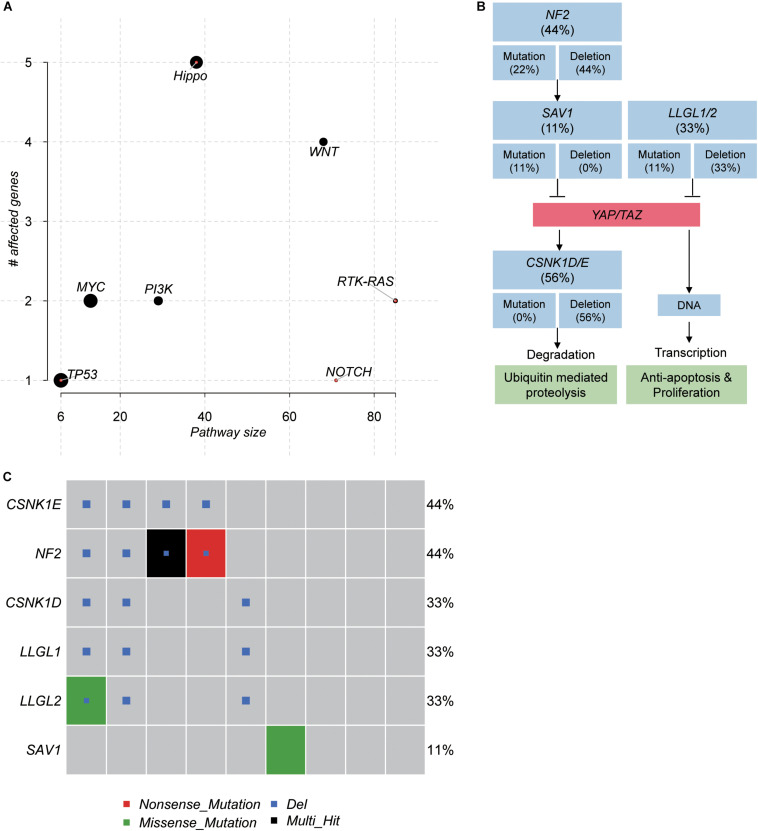
The pathways frequently mutated in spinal schwannoma. **(A)** The significance of the pathways frequently mutated in spinal schwannoma. The *y*-axis and *x*-axis represent the total number of genes and the number of mutated genes in the pathways. **(B)** The mutated genes and frequency in Hippo signaling pathway. **(C)** The number of mutated genes in Hippo signaling pathway across the nine spinal schwannomas. The *x*-axis represents the spinal schwannoma samples. The Multi_Hit indicates multiple variants of a gene were identified in the same sample.

To further investigate the critical pathways that controlled the initiation of schwannoma, we mapped the genes with somatic mutations to the oncogenic pathways. The Hippo signaling pathway was observed to be frequently mutated in schwannoma (Figure 4A). Combining the somatic mutations with CNAs, we found that *NF2*, *SAV1*, *LLGL1/2*, and *CSNK1D/E* were key regulators with mutations in Hippo signaling pathway (Figure 4B). Particularly, *NF2*, *SAV1*, and *LLGL1/2*, the upstream regulators of YAP/TAZ transcription factors, were frequently deleted or mutated with loss-of-function patterns, which may promote the transcription of YAP/TAZ target genes (Figure 4B). Among the 9 samples, 6 were detected to have mutations in the Hippo signaling pathway (Figure 4C), accounting for 67% of the samples. These results suggested that the Hippo signaling pathway was a critical pathway for schwannoma.

## Discussion

Spinal schwannoma is the most common primary spinal tumor, typically arising from spinal nerve roots. To investigate the genomic landscape of the spinal tumor, we performed whole genome sequencing of nine tumors and paired blood samples with high coverage (Supplementary Table S3). Specifically, we identified *TTN*, *MUC4*, *FLG2*, *MUC17*, *OR2T4*, *ZNF850*, *FAM186A*, *ALMS1*, *FAM47C*, and *ATM* as the top ten mutated genes. However, further analysis of those frequently mutated genes revealed that most of them were long genes and had lower VAF, indicating that most of these genes were passengers following the driver genes. Particularly, *ATM*, accounting for 33% of the samples, was the most frequently mutated cancer-related gene in schwannoma, followed by *CHD4*, *FAT1*, *KMT2D*, *MED12*, *NF2*, and *SUFU* (>20%). Notably, *SUFU* was reported to be responsible for meningioma (Aavikko et al., 2012), a type of tumor from central nervous system. In accordance with previous studies of sporadic schwannoma by next generation sequencing (Agnihotri et al., 2016; Havik et al., 2018), *NF2* was identified as the pathogenic gene in both spinal and vestibular schwannomas. As the other genes like *CHD4*, *FAT1*, *KMT2D*, and *MED12* were not detected in vestibular schwannoma, we thus speculated that some of them might be specific in spinal schwannoma.

Moreover, we also profiled the somatic CNA in schwannoma. *NF2* gene, harbored in the cytoband 22q11, was also identified to be frequently deleted in the samples of spinal schwannoma. The integrative analysis of the *NF2* point mutations, InDels and copy number deletions revealed that CHG004880 and CHG004884 had bi-allelic mutations in *NF2*, resulting in bi-allelic NF2 inactivation. Consistently, the occurrence of both somatic mutations and CNAs in *NF2* has been reported to result in schwannoma by previous studies (Jacoby et al., 1994; Rm et al., 2018).

Furthermore, we also investigated the genes within these CNAs in spinal schwannoma. We found that there were only a few genes located within the amplified regions. In contrast, the deleted regions contained a majority of genes (Supplementary Tables S1, S2). The higher frequency in deletions than gains suggested that the tumorigenesis of spinal schwannoma might be caused by these deletions. Particularly, losses of *XRCC2* and *MLL3/KMT2C*, which were located within 7q36.1, were also observed in the spinal schwannomas. *XRCC2* and *MLL3/KMT2C* were involved in homologous recombination repair and histone modification, suggesting that loss of DNA damage repair and epigenetic alterations might also play key roles in schwannoma.

Besides, among the somatic mutations and CNAs in the nine spinal schwannomas, *SMARCB1*, *SMARCE1* or *LZTR1* were absent in these spinal schwannoma tissues. As the familial spinal schwannoma usually have germline mutations in *SMARCB1*, *SMARCE1* or *LZTR1*, to our knowledge, these mutations are not prevalent in sporadic spinal schwannoma patients.

In addition, to identify genes potentially initiating the schwannoma, we ranked the genes with somatic mutations by the median of VAF across the samples (Figure 3A). Combined with the clonality analysis, *NF2* gene was a candidate driver in spinal schwannoma. Besides *NF2*, *SOX10* and *CHD4* also showed relatively high VAF among those genes (VAF > 20%). Notably, a previous study reported that loss of SOX10 function contributed to the phenotype of human Merlin-null schwannoma cells, indicating that the mutation of SOX10 might be associated with the initiation of schwannoma (Doddrell et al., 2013). Moreover, the different location of CHD4 staining has been reported to be used as a potential biomarker to differentiate cellular schwannoma from malignant peripheral sheath tumor (MPNST) (Wu et al., 2018). In addition, *NF1* and *CDKN2C* were also found to be homozygously deleted in spinal schwannoma. To our knowledge, *NF1* mutations were rarely reported in spinal schwannoma. Exceptionally, lack of NF1 expression has been observed in a sporadic schwannoma from a patient without neurofibromatosis (Gutmann et al., 1995), suggesting that *NF1* might be a novel driver in spinal schwannoma. Similarly, loss of *CDKN2C*, a cell growth regulator that controls cell cycle G1 progression, might also contribute to spinal schwannoma by the manner of CDKN2A (Koutsimpelas et al., 2011; Rohrich et al., 2016). Moreover, the pathway-level analysis revealed that Hippo signaling pathway was one of the frequently mutated pathways (6/9, 67%), in which, *NF2*, *SAV1*, and *LLGL1/2* were frequently deleted or mutated with loss-of-function patterns. *SAV1* and *LLGL1/2* have not been reported to cause the tumorigenesis of spinal schwannoma. However, the Hippo signaling pathway has been widely reported to be implicated in schwannoma by previous studies (Nikuseva-Martic et al., 2007; Oh et al., 2015; Brodhun et al., 2017; Zhao et al., 2018). *SAV1* promotes activation of MST-LATS kinase cascade in Hippo signaling, which suppresses the activities of YAP/TAZ by phosphorylation (Bae et al., 2017). *LLGL1/2* have been recognized as direct negative regulators of YAP/TAZ via phosphorylation (Cordenonsi et al., 2011). The loss of these tumor suppressors might lead to lower phosphorylation of YAP/TAZ, thereby promoting YAP/TAZ to translocate to the cell nucleus.

Admittedly, the present study still had some limitations. First, the sample size is small, which limited the accuracy of the conclusions. Second, the pathogenicity of the mutated genes in schwannoma need to be further validated. Third, the consequences of these potentially pathogenic genes and the underlying mechanisms should be further investigated. However, we aimed to identify the potentially pathogenic genes responsible for schwannoma, and the systematic analysis of DNA sequencing data improved our understanding of the genomic landscape in spinal schwannoma.

## Data Availability Statement

The raw data have been uploaded to the National Omics Data Encyclopedia (NODE) database (https://www.biosino.org/node/) with accession number OEP000894.

## Author Contributions

XG, WZ, ZL, and JX conceived and designed the study. LZ conducted the analyses. XG and QJ drafted the manuscript. LT, WG, TW, and ZW assisted with the analysis and the interpretation of the results. All authors read and approved the manuscript before submission.

## Conflict of Interest

The authors declare that the research was conducted in the absence of any commercial or financial relationships that could be construed as a potential conflict of interest.

## Supplementary Material

Supplementary Figure 1 The coverage and B allele frequency of chromosome 22 in samples of CHG004878, CHG004880, CHG004884, and CHG004888. The whole chromosome loss in chr22 was observed in samples CHG004878, CHG004880, and CHG004884, but the CHG004888 was found to have focal deletion adjacent to NF2. The position of NF2 in chr22 was highlighted by blue boxes.

Supplementary Figure 2 The read alignment of NF2 mutations in CHG004880 and CHG004884 by integrative genomic viewer (IGV).

Supplementary Figure 3 The high resolution version of Figure 3B.
