# Supplementary figures and images for: Whole Genome Sequencing Identifies Key Genes in Spinal Schwannoma
Source: Front Genet. 2020 Oct 30;11:507816. doi: 10.3389/fgene.2020.507816 (PMC7661748; doi:10.3389/fgene.2020.507816)

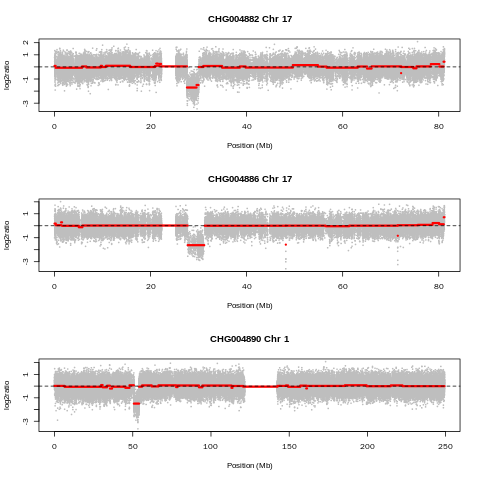

Supplement: Supplementary Figure 1 — The coverage and B allele frequency of chromosome 22 in samples of CHG004878, CHG004880, CHG004884, and CHG004888. The whole chromosome loss in chr22 was observed in samples CHG004878, CHG004880, and CHG004884, but the CHG004888 was found to have focal deletion adjacent to NF2. The position of NF2 in chr22 was highlighted by blue boxes. [file Image_1.TIFF]

A

CHG004880 (NF2: NM\_181832, c.762\_806del)

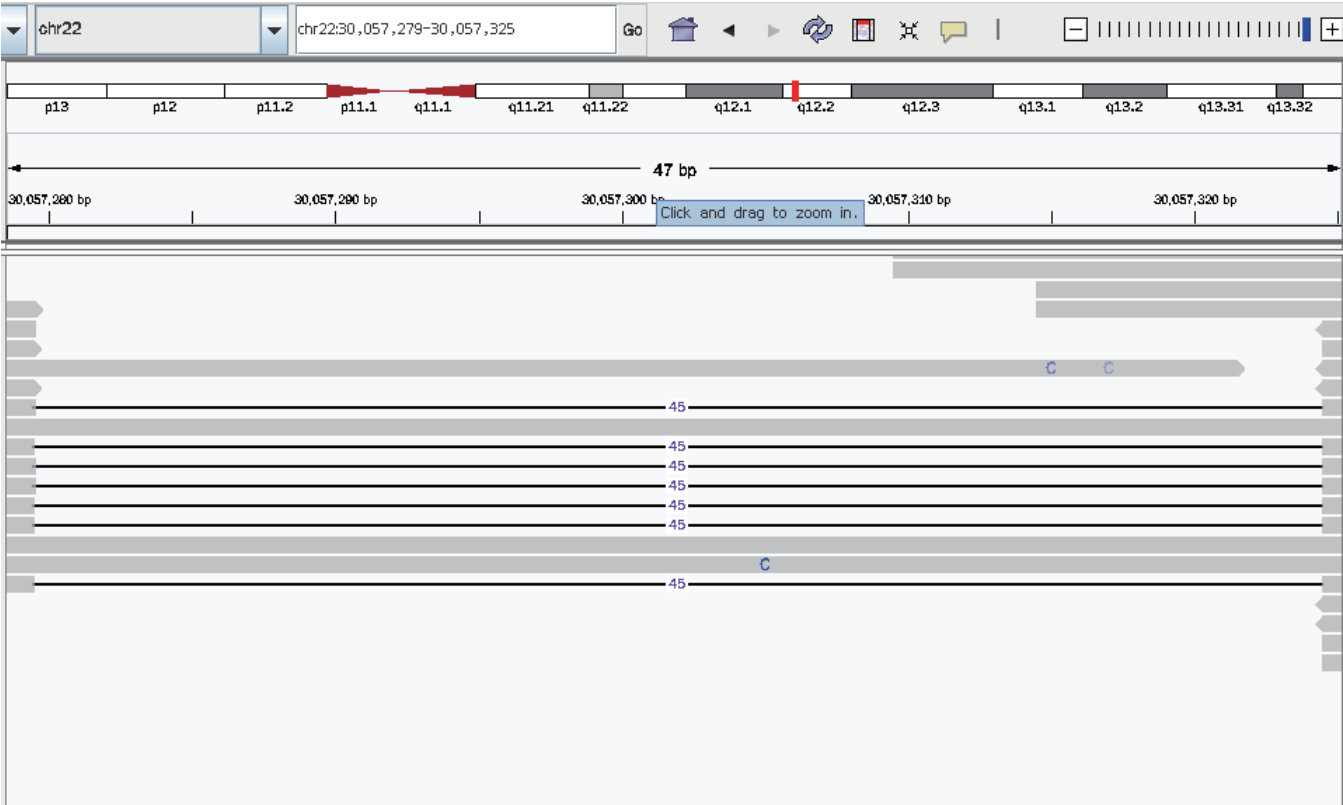

B

CHG004884 (NF2: NM\_181832, c.C1228T)

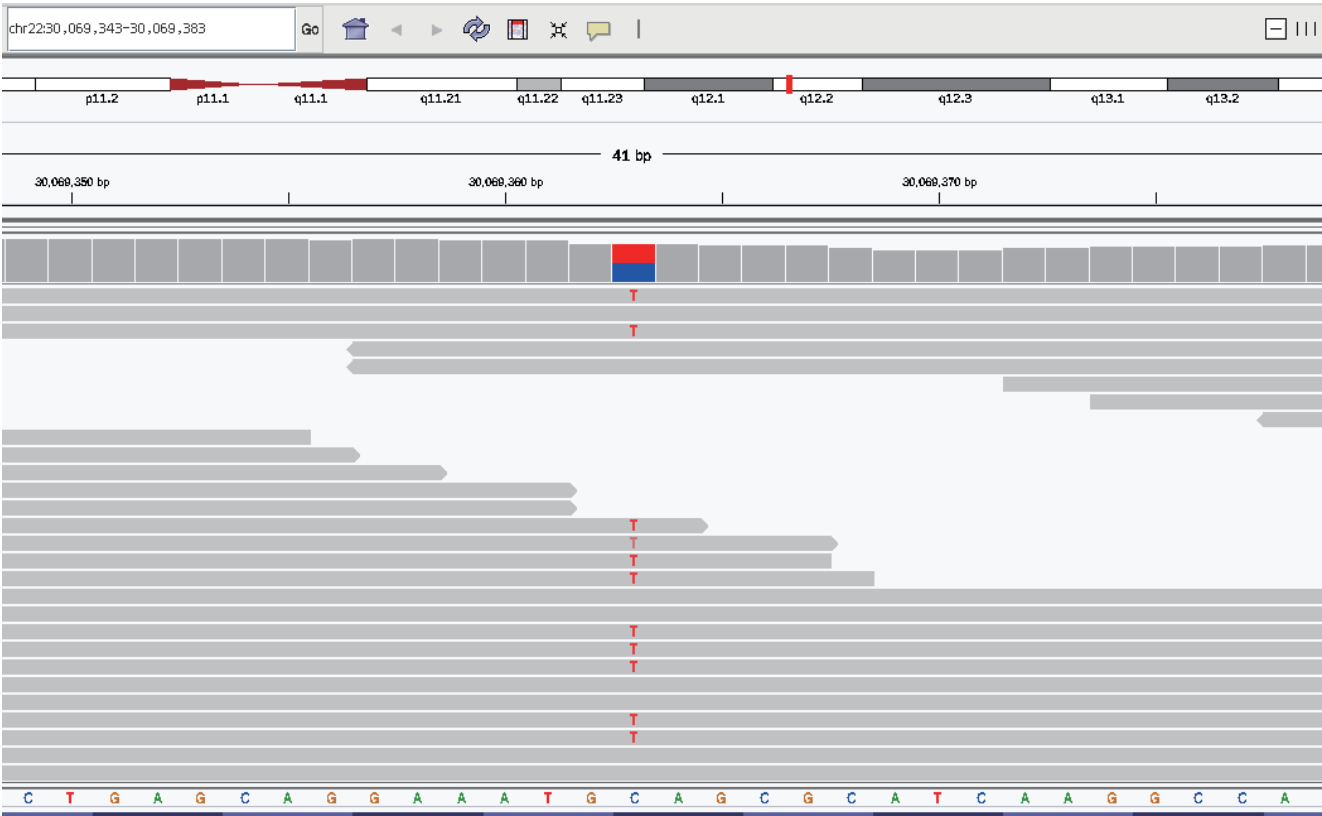

Supplement: Supplementary Figure 3 — The high resolution version of Figure 3B. [file Data_Sheet_2.PDF]
