# Supplementary material for: Whole Genome Sequencing Identifies Key Genes in Spinal Schwannoma
Source: Front Genet. 2020 Oct 30;11:507816. doi: 10.3389/fgene.2020.507816 (PMC7661748; doi:10.3389/fgene.2020.507816)
Supplement: Supplementary Figure 2 — The read alignment of NF2 mutations in CHG004880 and CHG004884 by integrative genomic viewer (IGV). [file Data_Sheet_1.PDF]

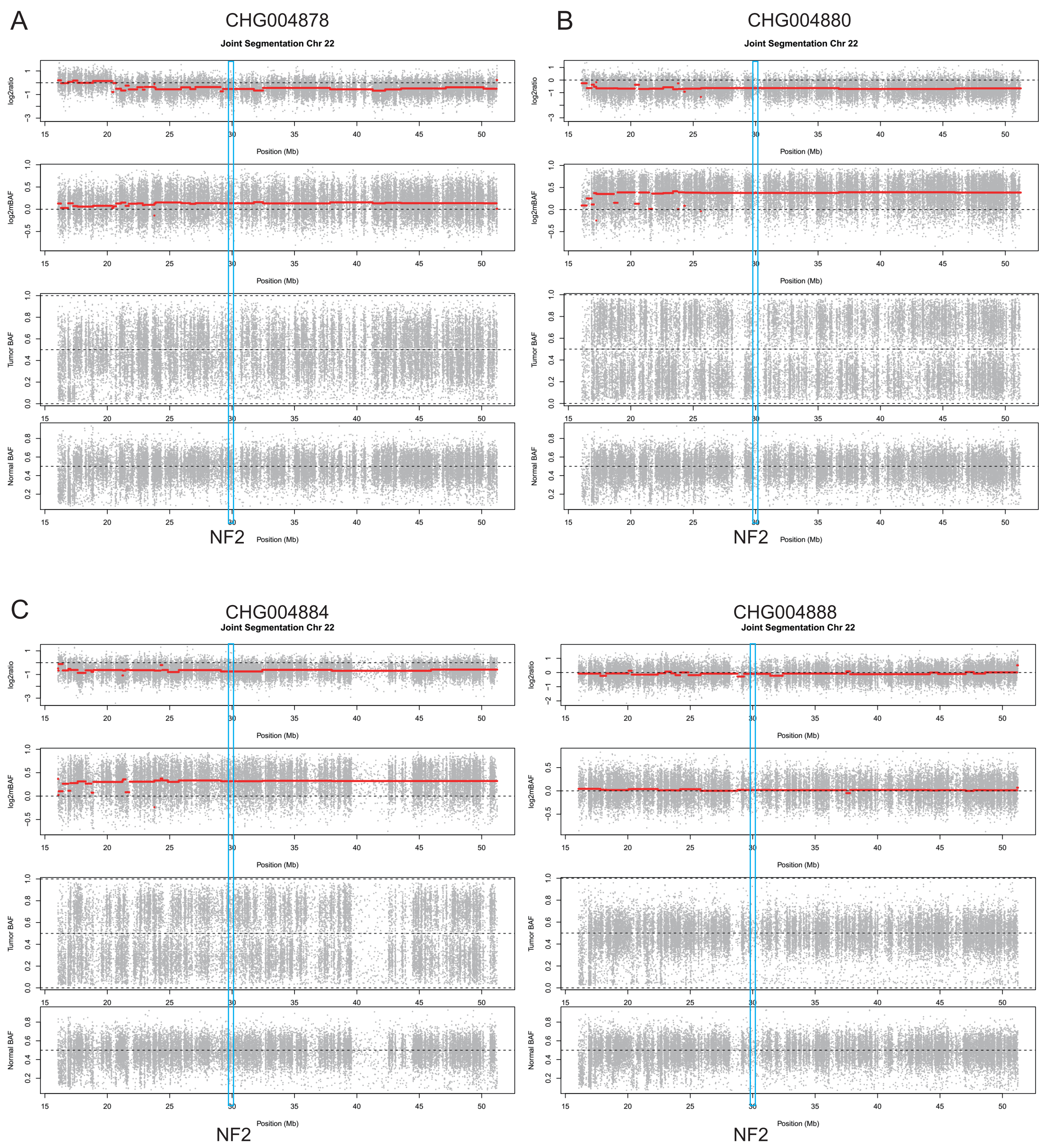
A
CHG004878
Joint Segmentation Chr 22
log2ratio
Position (Mb)
log2mBAF
Position (Mb)
Tumor BAF
Normal BAF
NF2
Position (Mb)
B
CHG004880
Joint Segmentation Chr 22
log2ratio
Position (Mb)
log2mBAF
Position (Mb)
Tumor BAF
Normal BAF
NF2
Position (Mb)
C
CHG004884
Joint Segmentation Chr 22
log2ratio
Position (Mb)
log2mBAF
Position (Mb)
Tumor BAF
Normal BAF
Position (Mb)
NF2
CHG004888
Joint Segmentation Chr 22
log2ratio
Position (Mb)
log2mBAF
Position (Mb)
Tumor BAF
Normal BAF
Position (Mb)
NF2
